# Induced Pluripotent Stem Cell Meets Severe Combined Immunodeficiency 

**DOI:** 10.22074/cellj.2020.6849

**Published:** 2020-09-08

**Authors:** Reza Kouchaki, Bahareh Abd-Nikfarjam, Amirhosein Maali, Saeid Abroun, Farshad Foroughi, Sasan Ghaffari, Mehdi Azad

**Affiliations:** 1. Faculty of Allied Medicine, Qazvin University of Medical Sciences, Qazvin, Iran; 2. Department of Immunology, School of Medicine, Qazvin University of Medical Sciences, Qazvin, Iran; 3. Student Research Committee, Pasteur Institute of Iran, Tehran, Iran; 4. Department of Hematology and Blood Banking, Faculty of Medical Sciences, Tarbiat Modares University, Tehran, Iran; 5. Hematology Department, School of Allied Medicine, Tehran University of Medical Sciences, Tehran, Iran

**Keywords:** Hematopoietic Stem Cell Transplantation, Induced Pluripotent Stem Cell, Primary Immunodeficiency, Severe Combined Immunodeficiency

## Abstract

Severe combined immunodeficiency (SCID) is classified as a primary immunodeficiency, which is characterized by impaired T-lymphocytes differentiation. *IL2RG, IL7Ralpha, JAK3, ADA, RAG1/RAG2*, and *DCLE1C* (Artemis) are the most defective genes in SCID. The most recent SCID therapies are based on gene therapy (GT) of hematopoietic stem cells (HSC), which are faced with many challenges. The new studies in the field of stem cells have made great progress in overcoming the challenges ahead. In 2006, Yamanaka et al. achieved "reprogramming" technology by introducing four transcription factors known as Yamanaka factors, which generate induced pluripotent stem cells (iPSC) from somatic cells. It is possible to apply iPSC-derived HSC for transplantation in patients with abnormality or loss of function in specific cells or damaged tissue, such as T-cells and NK-cells in the context of SCID. The iPSC-based HSC transplantation in SCID and other hereditary disorders needs gene correction before transplantation. Furthermore, iPSC technology has been introduced as a promising tool in cellular-molecular disease modeling and drug discovery. In this article, we review iPSC-based GT and modeling for SCID disease and novel approaches of iPSC application in SCID.

## Introduction

**Fig.1:**
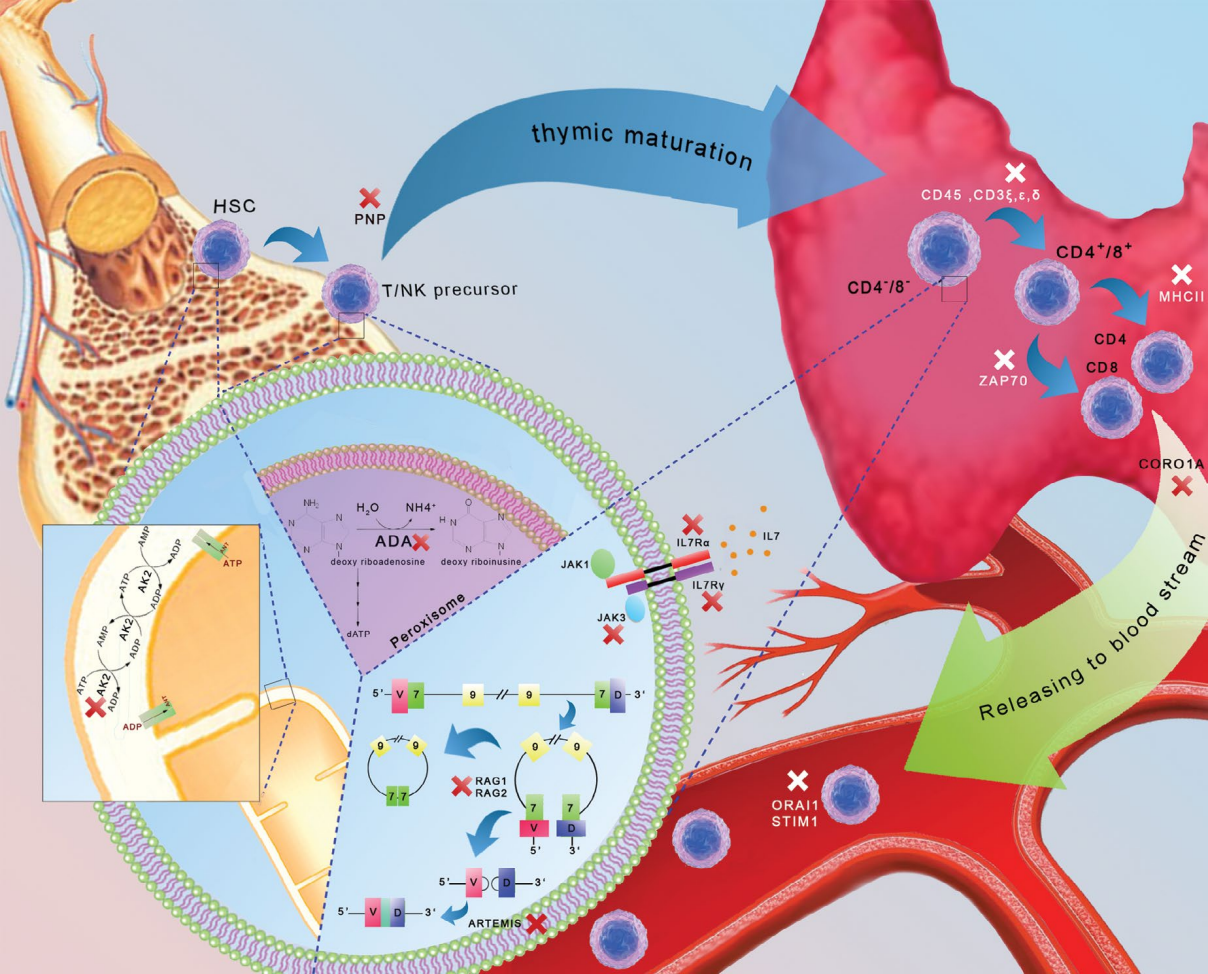
A depiction of the most common types of SCID. The deficiencies appear in different stages of T-cell development. AK2-deficiency, ADA-SCID, and PNP-SCID are caused by a deficiency in the developmental stages of the bone marrow. The defective signals from the pre-TCR and TCR (CD45, CD3ξ, CD3ε, CD3δ), ILR γ and α, RAG1/2, JAK3, ZAP70, MHCII, and ARTEMIS deficiencies occur in thymus developmental stages. SCID; Severe combined immunodeficiency, ADA; Adenosine-deaminase, PNP; Purine nucleo-side phosphorylase, and TCR; T-cell receptor.

Severe combined immunodeficiency (SCID) is classified as a primary immunodeficiency (PID), which is characterized by impaired T-lymphocyte differentiation. SCID is a monogenic, heterogeneous, and life-threatening syndrome (1). Considering that both humoral and cellular adaptive immunity are involved, this immunodeficiency is called "combined" because in T^-^-B^-^- phenotypes of SCID, T-cell development, as well as B-cell development is affected. In T^-^B^+^ phenotypes, the absence of normal T-helpers leads to defective antibody production by normal B-cells. In some subtypes of SCID, the disease can also be accompanied by defective natural killer (NK) cells. These different phenotypes are due to mutations in several genes, which lead to appear in different stages of T-cell development (Fig .1). The worldwide prevalence of SCID is estimated to be in 50,000 to 100,000 of the young population and constitutes 7% of PID patients. Approximately 90% of genetic defects in different forms of SCID have been identified (2, 3). The latest therapies regarding SCID are based on gene therapies (Table 1), which so far are faced with many difficulties (4-18). The new studies in the field of stem cells have made considerable progress in overcoming the challenges ahead.

### A review on induced pluripotent stem cell

In 2006, Takahashi et al. (19) achieved "Reprogramming" technology by introducing OCT4, KLF4, SOX2, and C-MYC reprogramming factors (RFs), which are responsible for embryonic-like state, into human fibroblasts. These RFs, known as OKSM factors, generate induced pluripotent stem cells (iPSC) from a somatic cell and reverse its state back into embryonic status, which can later differentiate to various human cells. IPSC-derived pre-differentiated or differentiated cells can be used for transplantation in patients with abnormal or poorly functional specific cell lineage. Considering that harvested cells are autologous, there is no risk of immunological rejection (fully matched HLA-profile) and no concern regarding the low number of transplantable cells. Furthermore, preparing these pluripotent stem cells is a non-invasive method (20).

In addition to other aspects of iPSC-based therapies, there are various studies in the field of cancer and immunodeficiency that led to the creation of iPSC-derived cytotoxic T-lymphocytes (iCTL) and iNKT-Cells, which have major roles in the immune system. The medical applications of iPSC are not limited to cell therapy. Recently, iPSC technology has been introduced as a promising tool for *in vitro* cellular-molecular disease modeling, drug discovery, and *ex-vivo* regenerative medicine, including organogenesis and GT (21, 22).

### The standard severe combined immunodeficiency treatments and challenges

Supportive care, such as intravenous immunoglobulin (IVIG) and antimicrobial agents, may be required for SCID. For example, cotrimoxazole is prescribed as prophylactic agent against *P. jirovecii*, acyclovir is used for patients with a history of herpes simplex virus infection, and antifungal prophylaxis should also be used. ADA-SCID can also be treated by enzyme replacement therapy (ERT), which consists of ADA coupled to polyethylene glycol (PEG-ADA), but the intramuscular injection is needed at least once a week to eliminate toxic waste. This is a life-saving therapy when other treatments are unavailable or less effective. This treatment is effective in approximately 90% of the patients. However, in some cases, after a period, the dose of PEG-ADA must be increased, mainly when anti-ADA antibodies have been produced. Systemic ERT may also ameliorate hepatic and neurologic dysfunctions in some ADA-SCID patients. A concern regarding ERT is lymphoid and possibly hepatic malignancies and progression of chronic pulmonary insufficiency. ERT before bone marrow transplantation may prevent donor cell engraftment by enhancing endogenous T-cell recovery (23).

Hematopoietic stem cell transplantation (HSCT) is the only definitive treatment for SCID and has more than a 90% chance of success (from geno-identical donors). There are some serious adverse effects, such as graft versus host disease (GvHD) or graft rejection, following the allogenic immune conflict between donor and recipient (24, 25). On the other hand, granulocyte colony-stimulating factors (G-CSF), such as filgrastim and lenograstim, which are used to release HSCs into the peripheral blood before leukapheresis, can also be mutagenic. However, chronic infection and thymus problems, which are related to the age of the recipient, can reduce the chance of success or cause damage to specific organs, like liver, kidney, or lung. In NKphenotypes of SCID, myeloablation or immunosuppression is not required for HSCT. NK cells can reduce the survival rate after transplantation of haplo-incompatible HLA. Due to the perpetual B-cell dysfunction, lifelong immunoglobulin substitution may be required to prevent infections caused by inadequate antibody responses (26).

**Table 1:** Review on common types of severe combined immunodeficiencies


Deficiency	Impaired gene	Locus	Inheritance pattern	Mechanisms	Lymphocyte profile	Frequency	Treatment	References

Signaling of IL7R	IL2RG	Xq13.1	XLR	Deficiency in common γ chain in IL7R and IL15R (associated with thymocytes and NK-cells differentiation signaling pathways, respectively)	T^-^B^+^ K^-^	48%	GT HSCT	(4-6)
	IL7R alpha	5p13	AR	A decrease in expression of IL7Rα on the surface of lymphocyte progenitor cells	T^-^B^+^NK^+^	10%	HSCT	(7, 8)
	JAK3	19p13.1	AR	Deficiency in the JAK3 tyrosine kinase	T^-^B^+^NK^-^	6%	HSCT	(9, 10)
Purine Metabolism	ADA	20q13.11	AR	The absence of ADA leads to accumulation of dAdo and dATP, which have functional and developmental deleterious roles in lymphocytes	T^-^B^-^NK^-^	16%	ERTGTHSCT	(11-13)
V(D)J Recombination	RAG1/RAG2	11p13	AR	Ceases the initiation stages of VDJ recombination process	T^-^B^-^NK^+^	6%	HSCT+Myeloablation	(10, 14)
	DCLE1C (Artemis)	10p	AR	Impairment in NHEJ (used for opening the hairpins during VDJ recombination) and disability of T-cell in rearranging TRG and TRB	T^-^B^-^NK^+^	5%	HSCT+Meloablation	(15-17)


AR; Autosomal recessive, XLR; x linked recessive, GT; Gene therapy, HSCT; Hematopoietic stem cell transplantation, ERT; Enzyme replacement therapy, SCID; Severe combined immunodeficiency, PNP; Purine nucleo-side phosphorylase, and TCR; T-cell receptor.

Transplantation from mismatched donors is still associated with high mortality. GT, as an alternative for haplotype HSCT procedure, is on clinical trials for both ADA-SCID and X-SCID, which will eventually determine the role of GT as a therapeutic option. The first vector that was used in GT was the γ-retroviral vector, which increases the expression of *MDS-EVI1* and *LMO2* (two insertional hotspots in HSC for γ-retroviral vector) and causes T-cell acute lymphoblastic leukemia (ALL) in some X-linked SCID patients (27- 29). Nevertheless, it has been used in ADA-SCID patients with a mild conditioning regimen, while no genotoxicity was seen. Self-inactivation (SIN) retroviral vector design has deletions in the U3 region of 5´LTR beside an internal heterologous promoter, which leads to the reduced incidence or even absence of proto-oncogene activation. Preclinical investigations on retroviral-mediated *JAK3* gene transfer shows expression of the exogenous JAK3 protein in animal models. SIN lentiviral vectors (LVs), which are based on the human immunodeficiency virus, have a better safety profile and reduce the risk of insertional mutagenesis. In addition, LVs are superior to γ-retroviral vectors in manipulating human HSCs and maintaining sustained transgene expression. SINlentiviral vector GT succeeded in preclinical murine models for ADA-SCID, RAG1-SCID, RAG2-SCID, and Artemis- SCID (30, 31). Considering all the challenges and therapeutic potentials of iPSC, the road to treating SCID seems clear, which ultimately leads to shorter and more efficacious treatment courses with fewer side effects.

#### Induced pluripotent stem cells-based gene therapy meets severe combined immunodeficiency

The invention of iPSC technology has allowed scientists to cure single-gene disorders by creating a healthy cell line from the patient in *ex vivo* conditions. After a biopsy of healthy cells, they are monitored in the laboratory. Lentiviruses and retroviruses were the first vectors that were used for transferring OKSM factors into somatic cells. The major problem with the use of these vectors is their mutagenicity, but lentiviral vectors are deemed to be less mutagenic than retroviral vectors (32). It has been shown that polycistronic lentiviral viruses, which have been combined with 2A self-cleaving peptides and internal Ribosomal Entry Site (IRES), are sufficient for the integration of RFs. Hence, the recombination of a polycistronic vector system with a lentiviral vector facilitates the clinical applications of iPSC. There are other methods to generate iPSC. These include non-integrating viruses, such as adenovirus, and non-viral approaches, such as plasmids, DNAdemethylating agents, histone deacethylating agents, and dimethyl transferases (33).

After the gene introduction, a particular time is required to allow the expression of factors and induction of pluripotency. Ultimately, iPSC gains immortality and self-renewal capacity (34). Direct iPSC-based cell therapy is used in non-hereditary diseases. In hereditary disorders, the iPSC-based cell therapy should be combined with GT to correct the defective gene(s). Due to the congenital deficiency in SCID, which is present in HSC, the primary step in iPSC-based treatment is generating “corrected” HSC. Each subtype of SCID results in different molecular mutagenic disorders. The most common ones are listed in Table 2. GT is used to modify these mutations and manipulate normal products by using iPSC-based GT.

There are many targeting vectors when genetically manipulating iPSC. These include classic targeting vectors, DSB-mediated targeting vector, BAC targeting vector, piggyBac targeting vector. Also, helperdependent adenovirus targeting vector, single-strand oligonucleotides (ssODN), and Adeno-associated virus (AAV) targeting vector are among advanced vectors. The nucleases bind to specific sites of DNA and catalyze it to a double-strand break (DSB). In the presence of donor DNA (targeting gene), homologous recombination occurs at a specific genomic site. The CRISPR-Cas9, meganucleases, zinc-finger nucleases (ZFN), transcription activator-like effector nucleases (TALEN) more recently have been engineered for this purpose. They decrease the dysregulation of gene expression and the off-targeting genotoxicity (40-43). After the correction of mutation, the iPSCs differentiate to HSCs, which is also influenced by specific hematopoietic cytokines and HOXB4 (44). In the next step, SCID patients are treated with induced HSCs transplantation with adequate dosage.

As discussed, many clinical trials have been performed in the field of iPSC-based GT. In 2007, Zou et al. (45) conducted the first iPSC-based GT for the treatment of sickle cell anemia. They reported the successful iPSC-based GT in sickle cell anemia in mice. There are other clinical trials, in iPSCbased SCID GT. In 2015, Chang et al. (46) attempted to correct JAK3 deficiency in SCID human iPSCs (hiPSC) using CRISPR/Cas9-enhanced gene targeting. It resulted in restoring normal T-cell development after gene correction. In 2015, Menon et al. (47) treated X1- SCID by generating the corrected iPSC line, which was modified by TALEN. Prior to this, in 2009, Lei et al. (48) differentiated T-Lineage from iPSCs and used it to treat RAG-deficient mice. In 2015, Howden et al. (49) modified reprogramming and gene correction by somatic cells of SCID patients using the CRISPRCas9 system. In 2016, Li et al. (50) edited IL-2RG locus in iPSC with a recombinant Adeno-associated virus (rAAV)-targeting vector. Despite the common problems in routine GT methods, there is no risk of immunological rejection and GvHD in iPSC-based GT (Table 3).

**Table 2:** Genetic alterations in common types of severe combined immunodeficiency


Types /number of mutations									
Defective gene	Gene size	Number of exons	Missense	Nonsense	Splice-site	Insertion	Deletion	Hotspot	References

*IL7R*	20738	8	3	1	1	-	-	In exon 4	(7)
*IL2RG*	5447	8	55	33	33	10	32	CG dinucleotides at cDNA 690-691 and cDNA 879	(4)
*JAK3*	24029	25	13	7	3	1	2	-	(35)
*RAG1*	12544	2	36	10	-	1 (frameshift)	12 (frameshift)	-	(36)
*RAG2*	7092	2	15	2	-	1 (frameshift)	1 (frameshift)1 (in-frame)1 (gross)	-	(37)
*ARTEMIS*	56665	18		1	3	-	3 (frameshift)	-	(38)
*ADA*	33003	10	37	3	9	-	4 (frameshift)1 (in-frame and gross)	-	(39)


**Table 3:** Current studies on iPSC-based trials on severe combined immunodeficiency


Year	Scientists	Disease	Type of investigation	Origin of iPSC	Reprogramming vector	Reprogramming factor	Gene editing vector	Results	References

2009	Lei F et al.	RAG-SCID	Modeling	Mouse embryonic fibroblast (MEF)	Retroviral	OCT3/4, SOX2, KLF4, c-MYC	-	The first report of T-cell generation from iPSCs (iPSCs were converted into T-cells, which contained TCRβ and CD3. T-cells were stimulated by anti-CD3 and anti-CD28 antibodies to secrete IL2 and INFγ. Then, by transferring them into RAG-SCID mice, it led to the reconstituting of T-cell pools).	(48)
2015	Chang CW et al.	JAK3-SCID	Modeling(+Gene editing)	Keratinocyte	lentiviral	OCT4, SOX2, KLF4, c-MYC	CRISPR/Cas9	The iPSC was derived from a JAK3-SCID patient and showed that the differentiation of T-cells that contained this defect stops at early developmental stages. The correction of JAK3-mutation via CRISPR/Cas9 led to regular development and production of mature NK and T-cells with a broad TCR repertoire.	(46)
2015	Howden SE et al.	ADA-SCID	Gene editing	Fibroblast	episomal	OCT4, SOX2, KLF4, c-MYC, NANOG, LIN28,SV40 large T-antigen	CRISPR/Cas9	Reprogramming and gene targeting in a one-step process significantly reduce the time and resources, as well as the risks of cell cultures, drug selection, and multiple clonal events.	(49)
2016	Menon T et al.	X-SCID	Modeling (+gene editing)	BM-MSC (bone marrow mesenchymal stem cell)	lentiviral	-	TALEN	The mutant X1-SCID iPSC can produce hematopoietic and myeloid precursors, but the wild type and the gene-corrected iPSC can also provide mature NK and T-cell precursors with healthy IL2Rγ production.	(47)
2016	Li LB et al.	X-SCID	Modeling (+gene editing)	MSCs (mesenchymal stem/stromal cells)	Lentiviral	OCT4, SOX2, NANOG, LIN28	rAAV	Illustrated the role of IL2RG in the developmental evolution of NK-cells and T-cells, and the application of correcting IL2RG mutation in iPSC in regenerative medicine to prevent GvHD.	(50)
2015	Brauer PM et al.	RAG1-SCID (and RAG1-OS)	Modeling	Modeling	Lentiviral	OCT4, SOX2, KLF4, c-MYC	-	Evaluation and comparison of T-cell development, along with TCR V(D)J recombination in OS and SCID patients, and showing the correlation of genotype-phenotype in different patients with different mutations in the same gene.	(51)


SCID; Severe combined immunodeficiency and iPSCs; Induced pluripotent stem cells.

#### Severe combined immunodeficiency modeling based on induced pluripotent stem cells

In addition to all its clinical benefits, iPSC technology is also used in the human disease modeling to identify the exact genomic and molecular pathological pathways of a disorder. It is also used in drug discovery to design efficient, safe, and novel drugs and screen their efficacy and toxicity. The use of animals for human disease modeling have ethical issues, limitation in completely resembling human disease phenotypes due to fundamental differences between human and animal genomes. Furthermore, the inaccessibility to animals and difficult preparation (impossible in some cases) of specific cell-line *in vitro* makes this method more problematic.

In contrast, patient-derived iPSC has enabled scientists to provide high numbers of disease-specific cell-lines in laboratory conditions and help them overcome the mentioned problems. Moreover, healthy iPS cells, which are derived from the patient, can be used as a control in the modeling process. As mentioned, the accurate recognition of monogenic mutations is a key step in understanding the pathogenesis of the disease (52). Consequently, the action to reverse this mutation is a major step in the treatment of genetic disorders. The *in vitro* modeling of gene editing is an introduction to the application of *in vivo* GT. There are conventional tools for gene modification that are used in GT in clinics and modeling. Among the programmable site-specific nucleases, CRISPR-Cas9 system has been highly regarded for its ability to create a wide range of isogenic controls for iPSC-based modeling and its simplicity in design and use (53).

Since the inception of this technology, many studies have been conducted based on iPSC-disease modeling, such as Ciliopathy, Parkinson’s disease, hematopoietic abnormalities, cardiac disorders, insulin resistance, and metabolic syndrome, skeletal muscle disorders, schizophrenia pathogenesis, amyotrophic lateral sclerosis, mitochondrial disorders, etc. (54-56). The use of iPS cells in the modeling of primary immunodeficiencies, such as chronic granulomatous disease (CGD) and SCID, has also been successful (19).

**Fig.2:**
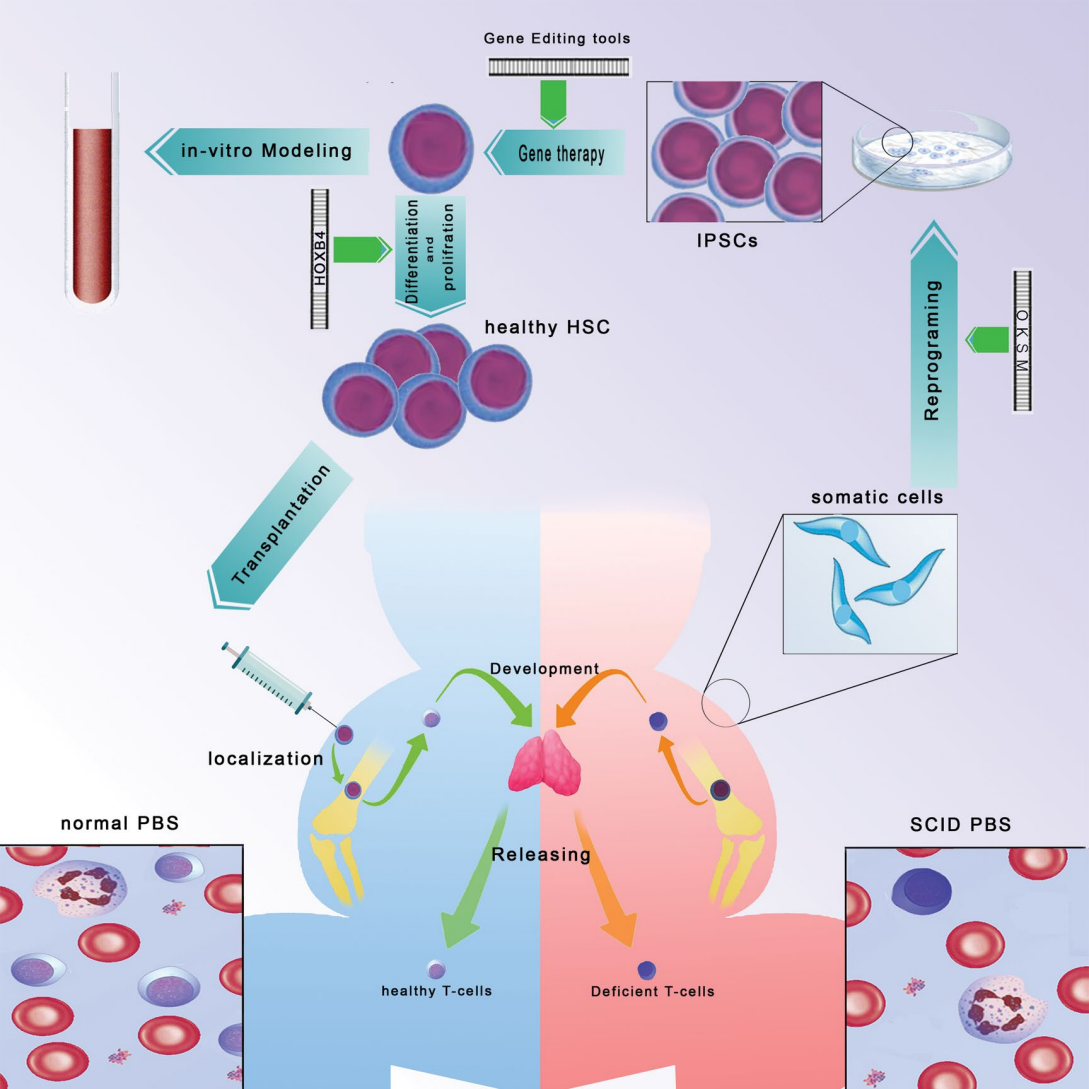
A review of iPSC technology applications in SCID. OKSM RFs reprogram somatic cells to iPSCs. Hematopoietic RFs are used to induce hematopoiesis in iPSCs. The prepared cells are used for *in vitro* modeling and HSCT (after GT). SCID; severe combined immunodeficiency, OKSM; OCT4, KLF4, SOX2 and c-MYC, RFs; Reprogramming factors, iPSCs; Induced pluripotent stem cell, HSCT; Hematopoietic stem cell transplantation, and GT; Gene therapy.

In 2008, one of the first PID models based on iPSC was done by Park et al. (57). In 2015, Chang et al. (46) recognized that the Jak3-deficient T-cell progenitor development is blocked in early stages by using iPSCs derived from SCID patients. In this study, gene editing by the CRISPR-Cas9 system restored the development of early T-cell progenitors. These modified progenitors could differentiate into healthy NK cells and T-cells. In 2016, Brauer et al. (51) modeled T-cell development by iPSCs from RAG1-SCID patients. They recognized that *RAG1* mutation has low recombination capability and results in cleavage defects. These studies are powerful tools for identifying the PID mechanisms, pharmacological tests, and GT trials (Fig .2). There are other applications of iPSCs in the field of immunodeficiency (Table 4).

#### Induced pluripotent stem cell application in secondary diseases of severe combined immunodeficiency and another sight

IPSC can also generate B-cells in T-B- phenotypes of SCID. In advanced SCID, secondary diseases are often seen. In addition to the primary treatment of SCID that restores the immune system, the application of regenerative iPSC technology can also be used for secondary diseases of SCID. Sensorineural hearing loss (SNHL), which is the result of reticular dysgenesis progression, is caused by damage or deficiency in cochlear, which is followed by SCID.

Moreover, deterioration of bone (leading to costochondral dysplasia), thymic epithelium, lung, liver, and brain tissues results in progressive ADA-SCID. PNP-SCID causes neurological abnormalities. RHOH deficiency induces Burkitt lymphoma. ORAI1 and STMI1 deficiencies lead to non-progressive myopathy and ectodermal. MATG1 deficiency can also cause neoplasia (70). Most of the aforementioned disorders are characterized by specific cell-line deficiency. IPSC-derived cells, prepared *in vitro*, can replace faulty cell-lines. In organopathies, there are clinical approaches to organogenesis and histogenesis based on iPS cells, which were inaccessible before. Malignancies and other disorders are also in the context of iPSC technology. DiGeorge syndrome, characterized by the impaired thymus, leads to SCID. The standard treatment for DiGeorge syndrome is allograft thymus transplantation. The risk of immunological graft rejection can be eliminated by iPSC regenerative technology. Therefore, iPSC appears to be a suitable and comprehensive therapeutic option for SCID patients.

SCID is a fatal PID characterized by impairment in T-cells development. Standard therapeutic plans for SCID are not safe. In grafted cases, there is a risk of GvHD and immune rejection due to the impairment in the immune system. The Side effects of ERT and myeloablation cause a systemic defect in patients. Altogether, we do not have access to optimal therapy for SCID yet.

We discussed different aspects of a critical key in SCID treatment in the future: iPSC therapy, which potentially is optima for SCID therapy. However, many challenges are facing to iPSC technology. As discussed, it could be achieved to the high-pure cell products based on iPSC, including iCTL, iNK cells, HSC, etc., making a safe procedure to transplantation (no immune rejection, no GvHD) as an autologous graft in SCID patients. However, due to the pluripotency state of iPSCs, there is a teratogenesis risk that limited clinical administrations of iPSCs for now. However, there are some reports to overcoming on teratogenesis of iPSCs and entrance in clinical trials. So, iPSC is a promising window for "Bubble boys" in the future, not so far.

**Table 4:** Current studies on iPSC-based trials on other immunodeficiency disorders


Disease	Year	Conducted by	Type	Results	References

AIDS	2010	Kamata et al.	Modeling (+gene editing)	Application of chimeric MLV (murine leukemia virus)/lentiviral vectors for iPSCs reprogramming (to take advantage of both vectors) and CCR5 shRNA (short-hairpin RNA) as an anti-HIV factor in GT based on iPSCs.	(58)
	2011	Kambal et al.	Modeling (+gene editing)	Generation of the immune cells derived from iPSCs that are resistant to HIV-1 via CCR5 shRNA and human/rhesus chimeric TRIMα gene as a pre-integration inhibitor.	(59)
	2012	Yao et al.	Modeling (+gene editing)	Destruction of CCR5 locus in hiPSCs via ZFN to generate healthy hematopoietic colonies in AIDS treatment.	(60)
	2015	Kang et al.	Modeling (+gene editing)	Destruction of CCR5 locus in hiPSCs via CRISPR/Cas9 to generate healthy hematopoietic colonies. The iPSCs-derived macrophages are resistant to CCR5-tropic viruses.	(61)
CGD	2011	Mukherjee et al.	Modeling (+GT)	Generation of iPSCs from X-CGD mice and comparison of the differentiation potential of these iPSCs to myeloid precursors and neutrophil compared to wild type iPSCs. GT by transferring gp^91phox^ and using a lentiviral vector regenerates the activity of NADPH oxidase in X-CGD iPSC-derived neutrophils.	(62)
	2011	Zou et al.	Modeling(+Gene editing)	Reproducing the pathognomonic CGD oxidase negative phenotype to show ROS deficiency in neutrophils derived from X-CGD iPSCs, and gp^91phox^ correction via ZFN and restoration of ROS production in neutrophils.	(63)
	2012	Jiang et al.	Modeling	The iPSCs generation from X-CGD and AR47-CGD patients, differentiation to monocytes, and macrophages by a similar cytokine profile to blood-derived macrophages. These macrophages have typical phagocytic properties but lack ROS production.	(64)
	2014	Brault et al.	Modeling	Presentation of the optimized protocols to generate macrophages and neutrophils derived from iPSCs in three types of CGD patients (i.e., x^0^-linked, AR47^0^, and AR22^0^for the first time).	(65)
	2015	Laugsch et al.	Modeling (+gene editing)	Restoration of NADPH oxidase activity in X-CGD iPSCs after differentiation to neutrophils in two ways: transposon-mediated integration of a BAC vector carrying the CYBB gene, and the correction of mutation via homologous recombination.	(66)
	2017	Brault J et al.	Modeling	The research on enzyme therapy by recombinant NOX2/p^22phox^ liposomes in macrophages derived from X-CGD iPSCs that led to the successful delivery of NOX2 and p^22phox^ to the plasma membrane and regeneration of NADPH oxidase complex and production of superoxide anions.	(67)
WAS	2015	Suphapeetiporn et al.	Modeling (+GT)	Correction of pro-platelet structures by overexpression of WASp in WAS iPSCs via lentiviral vectors.	(68)
	2016	Laskowski et al.	Modeling (+gene editing)	Correction of WASp in WAS-iPSCs via ZFN that led to the expression of WASp in all of hematopoietic lineages and repair of T-cells and NK-cells defect.	(69)


AIDS; Acquired immunodeficiency syndrome, CGD; Chronic granulomatous disease, WAS; Wiskott-Aldrich syndrome, iPSC; induced Pluripotent stem cell, and GT; Gene therapy.

### Conclusion

The technology of iPSC demonstrates a promising future in clinical applications. Since its invention, there is extensive research regarding various reprogramming methods to achieve iPSC. Many researchers have studied its differentiation, application in identifying disease pathogenesis, drug design, histogenesis, organogenesis, and cell therapy. The combination of iPSCs and GT has expanded its therapeutic potential and other aspects of this technology. Most of the clinical applications of iPSC, such as application in SCID, are still in the study phase. Although its introduction to the clinic is not far. As technology is increasingly used by scientists, the treatment of various diseases by iPSC technology is close.
